# Genomic advances in orphan and underutilized Brassicaceae crops and their wild relatives

**DOI:** 10.3389/fpls.2026.1878836

**Published:** 2026-06-18

**Authors:** Junrey Amas, Rajesh Kumar Natarajan, William J. W. Thomas, David Edwards, Jacqueline Batley, Aria Dolatabadian

**Affiliations:** 1 School of Biological Sciences, The University of Western Australia, Perth, WA, Australia; 2 Centre for Applied Bioinformatics, The University of Western Australia, Perth, WA, Australia; 3 The UWA Institute of Agriculture, The University of Western Australia, Perth, WA, Australia

**Keywords:** Brassicaceae genomics, crop diversification, molecular breeding, orphan crops, underutilized species

## Abstract

Orphan Brassicaceae crops, alongside other underutilized species and their wild relatives, represent an important but underexploited reservoir of genetic diversity with potential to enhance crop resilience, nutritional quality, and sustainable agriculture. These taxa include emerging oilseed, leafy vegetable, industrial, medicinal and stress-adapted species that harbor valuable traits such as tolerance to abiotic stresses, resistance to pests and diseases, improved seed and oil quality, and specialized bioactive compounds. Despite their potential, many of these species have received limited investment in breeding and genetic improvement compared to major Brassica crops. Recent advances in genomics and high-throughput sequencing have accelerated the development of genomic resources across Brassicaceae species, including chromosome-level genome assemblies, pan-genomes, and transcriptomic atlases. These resources have provided insights into genome evolution, gene family expansions, sub-genome dominance, and regulatory networks underlying key adaptive and agronomic traits. The increasing availability of genomic datasets has enabled molecular breeding approaches such as marker-assisted selection, genome-wide association studies, genomic selection, and genome editing. The close evolutionary relationships among Brassicaceae species further facilitate the transfer of knowledge and introgression of beneficial alleles from orphan and underutilized crops, as well as wild relatives, into cultivated Brassica species. Integration of genomic, transcriptomic, and multi-omics datasets enables the identification of candidate genes and regulatory pathways, guiding targeted breeding and accelerating the development of climate-resilient, nutrient-dense crops. This review highlights recent progress, challenges, and prospects for genomics-enabled breeding in orphan and underutilized Brassicaceae crops and their wild relatives.

## 1 Introduction

Global agriculture has experienced significant homogenization, with more than half of human caloric intake coming from just three cereal crops: rice, maize and wheat (Ye and Fan, 2021), and 70% of global calories derived from only 15 crop species (Khoury et al., 2014; Chang et al., 2019; Dawson et al., 2019). While this reliance has improved energy availability in diets, it has also created a fragile food system vulnerable to climate-related production shocks (Bailey et al., 2015), contributed to widespread hidden hunger (von Grebmer et al., 2014), and hastened the depletion of planetary resources (Rockström et al., 2009). The environmental impacts of intensive crop production systems, which rely heavily on chemical inputs, have further compromised sustainability, resulting in inefficient long-term productivity trajectories (Fernie and Yan, 2019). With the human population projected to reach 9.8 billion by 2050 (United Nations, 2025) and climate change worsening drought and heat stress, the need to diversify agricultural systems has never been more urgent.

Orphan crops are locally significant but under-researched species, typically cultivated in restricted regions with minimal international trade. While often grouped with broader categories of underutilized, minor or neglected crops (Tadele, 2019), orphan crops are distinguished from major crops by their limited research investment and marginal presence in global agricultural systems. They are broadly defined as species grown in restricted, often disadvantaged areas, and include numerous fruits, vegetables, legumes, grains, roots and tubers. For example, a significant portion of the undernourished population in Sub-Saharan Africa relies on crops such as sorghum, millets, tef, and various roots and tubers for their calorie requirements (Review of CGIAR Priorities and Strategies, 1994). Despite their importance in maintaining food security in regional communities, these crops have attracted relatively limited scientific focus (Varshney et al., 2012; Chiurugwi et al., 2019). Furthermore, many orphan crops are adapted to marginal environmental conditions, meaning they exhibit improved tolerance to stresses including salinity, drought, and heat stress (Gruber, 2017; Talabi et al., 2022). They can also be highly nutritious (Stadlmayr et al., 2013, 2019), and provide environmental benefits while filling multiple production niches (Dawson et al., 2019). Many of these crops utilize efficient C4 photosynthesis pathways and show resilience under low-input conditions, offering more sustainable options in areas where major crops would otherwise struggle (Ye and Fan, 2021). As a result, orphan crops represent valuable resources for diversifying food production and creating more resilient, sustainable and nutritious production systems (Frison et al., 2006; Mabhaudhi et al., 2016; Dawson et al., 2019).

Advances in genomics have underpinned unprecedented crop improvement through genome-based breeding and also offer avenues for the accelerated improvement of orphan and underutilized crops, as well as the utilization of wild relatives (Mohd Saad et al., 2022). Recent breakthroughs in genetics and genomics have uncovered significant similarities among plant genomes in gene content, biochemical pathways, and chromosome organization (Devos and Gale, 2000; Bennetzen, 2002), allowing knowledge transfer from extensively studied species to less-studied crops (Lemmon et al., 2018; Ye and Fan, 2021). For example, 99% of proteins in maize, wheat, and barley are also present in rice, and over 80% of Arabidopsis genes have corresponding genes in rice (Bennetzen, 2002). Currently, the genomes of at least 12 orphan cereals and their wild relatives have been sequenced, supplying valuable genetic resources for breeding and functional studies (Ye and Fan, 2021). Similarly, advances in genomics have significantly enhanced the understanding of Brassicaceae genomes, enabling the identification and characterization of genetic variation across major and orphan crops, underutilized species, and wild relatives. These developments pave the way for genomics-assisted breeding strategies, including marker-assisted selection (MAS) and targeted genome editing techniques, such as CRISPR/Cas9, to rapidly enhance genetic gain. These technologies offer promising avenues to accelerate crop improvement without requiring the same level of investment as traditional breeding approaches (Fernie and Yan, 2019; Jamnadass et al., 2020).

The Brassicaceae is a large angiosperm family, also known as the mustard family, comprising 338–360 genera (Anjum et al., 2014) and 4636 species (Francis et al., 2021; Hendriks et al., 2023). It is the largest family within the Brassicales order, which has a complex evolutionary history shaped by biochemical innovation, whole-genome duplications (Schranz and Mitchell-Olds, 2006; Barker et al., 2009) and dynamic biogeographical processes (Couvreur et al., 2010; Huang et al., 2016). The family constitutes some of the most globally important crops cultivated as oilseeds (*Brassica napus*), vegetables (*B. rapa* and *B. oleracea*), condiments/spices (*B. nigra* and *B. juncea*) and forage crops (*B. juncea* and *B. rapa*). In addition to these major crops, the Brassicaceae includes a diverse range of orphan crops, underutilized species, and wild relatives that serve as important genetic resources for crop improvement. These include orphan crops such as *Brassica carinata*, *Crambe abyssinica, Physaria fendleri, Pugionium cornutum* and *Lepidium meyenii*, underutilized species such as *Armoracia rusticana*, *Camelina sativa*, *Crambe hispanica*, *Diplotaxis tenuifolia, Diplotaxis muralis, Diplotaxis harra, Diplotaxis erucoides, Eruca sativa*, *Isatis indigotica*, *Lepidium sativum*, *Orychophragmus violaceus, Sinapis alba*, and *Thlaspi arvense*, and wild relatives such as *Camelina neglecta, Camelina microcarpa, Cardamine enshiensis, Hirschfeldia incana, Schrenkiella parvula*, *Barbarea vulgaris, Rorippa indica, Sisymbrium irio, Subularia aquatica*, and *Subularia monticola*. These species collectively possess valuable traits, including high nutritional quality, diverse phytochemical composition, and suitability for food, feed, and biofuel production. They also exhibit stress tolerance, pest and disease resistance, and specialized adaptive features such as enhanced photosynthesis and perennial growth, making them important resources for crop improvement. Their close evolutionary relationships with cultivated Brassica crops facilitate comparative evolutionary studies and the transfer of knowledge between species. By characterizing the genetic architecture of important traits in these taxa, breeders can not only improve orphan and underutilized crops but also identify homologous loci for improvement of major crops. However, as with many underutilized plant species, investment in these Brassicaceae crops has significantly lagged behind that in major crops, hindering their widespread utilization in agricultural production (Smil, 2000).

While orphan crops form the core focus of this review, it is important to distinguish them from the broader category of underutilized crops and from wild relatives. Underutilized crops may be cultivated beyond local scales but remain insufficiently exploited due to limited breeding investment or market development, whereas wild relatives are non-domesticated species that serve as important reservoirs of genetic diversity (Ye and Fan, 2021; Tirnaz et al., 2022). In the Brassicaceae, these categories form a continuum of utilization, ranging from locally cultivated orphan crops to emerging underutilized species and non-cultivated wild taxa. This distinction provides a conceptual framework for understanding their respective roles in crop improvement and genomic research. This review explores genomic advances in orphan and underutilized Brassicaceae crops and their wild relatives, and how these can be harnessed to improve both emerging and established crops. We first examine the diversity and importance of these species, and provide an overview of available genomic, transcriptomic and epigenomic resources. We then discuss how these resources support crop improvement through molecular breeding, comparative genomics and multi-omics approaches. Finally, we highlight key challenges and future opportunities for genomics-enabled breeding in the Brassicaceae.

## 2 Diversity and importance of orphan, underutilized and wild Brassicaceae species

**Figure 1:**
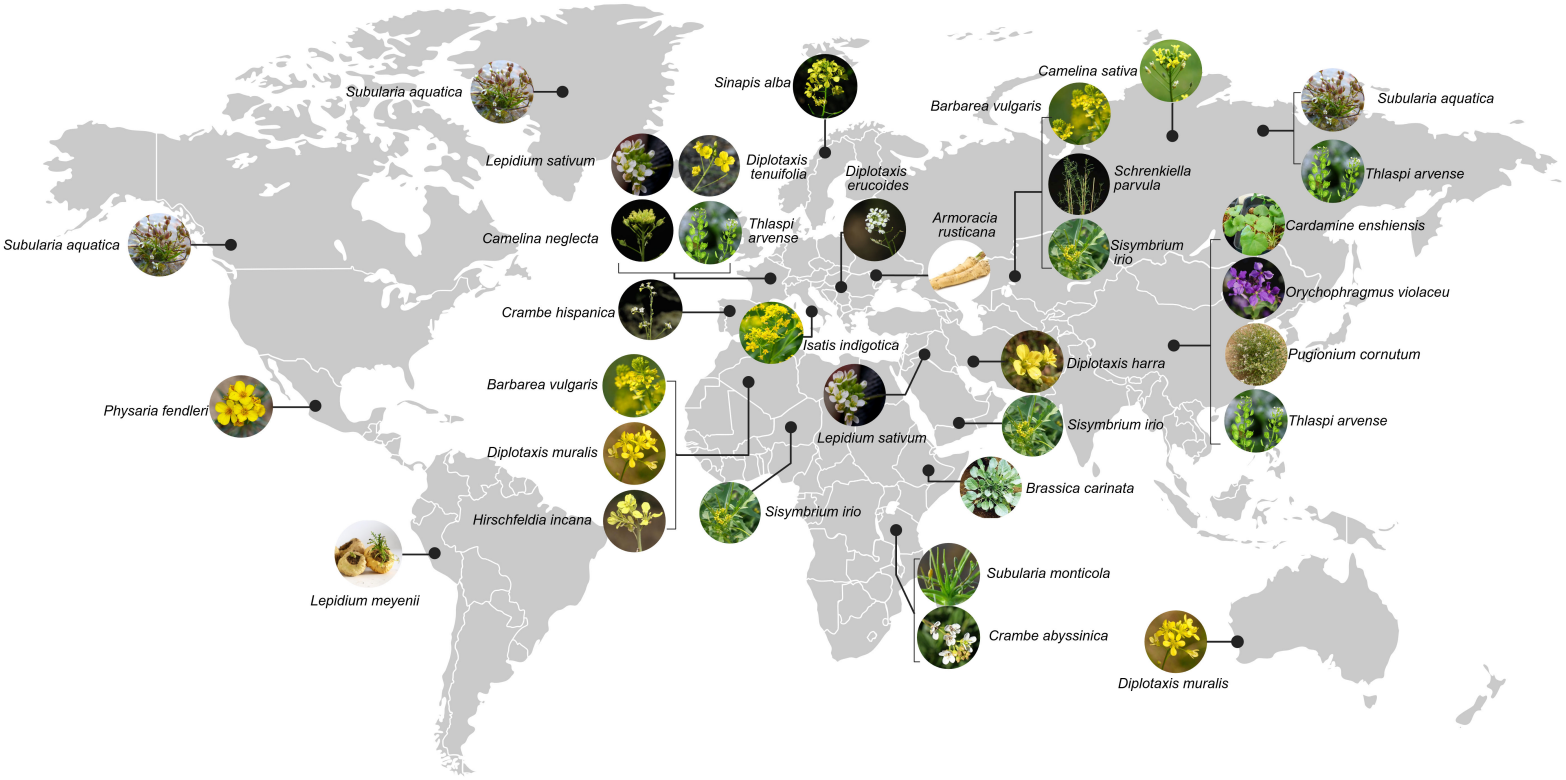
Global distribution of orphan Brassicaceae crops based on their native origin. Created with BioRender.com.

Out of the 66 plant families comprising 268 species represented as major food crops, the Brassicaceae family accounts for 15 species (Raza et al., 2020). In addition to these globally important crops, the family includes a diverse range of orphan crops, underutilized species, and wild relatives distributed across a wide range of ecological regions. This diversity reflects extensive adaptive evolution and highlights the importance of these species as both emerging crops and valuable genetic resources for crop improvement (**Figure 1**; **Table 1**).

**Table 1:** Representative traits in orphan, underutilized Brassicaceae species and their wild relatives.

Trait type	Trait description, representative species	Potential causal genes/pathways	Breeding Potential
Abiotic stress tolerance	Drought tolerance - *Pugionium cornutum* (Xu and Wang, 2024)	*PcHsp70-5*	Improving stress resilience under climate change
	Salinity tolerance - *Schrenkiella parvula* (Wijesinghege et al., 2022)	various genes including *PYRROLINE-5-CARBOXYLATE SYNTHETASE 1 (P5CS1)*	
	Flood resilience - *Thlaspi arvense* (Combs-Giroir et al., 2024)	various genes including *SUS1, HRE2, ATPS2, RBOHD, and SAG14*	
Biotic Stress Resistance	Pest resistance Diamondback moth - *Barbarea vulgaris* (Wei et al., 2013)	various genes including *β-AS, UDP-GLYCOSYLTRANSFERASES AND CYTOCHROME P450*	Reduces pesticide use and improves yield stability
	Aphid - *Rorippa indica* (Bandopadhyay et al., 2013)	several endo-chitinases, chitin-induced plant U-box type E3 ubiquitin ligases Not yet resolved; SSR and SNP markers available for germplasm characterization and future mapping of resistance loci	
	Fungal resistance – *Diplotaxis tenuifolia* (Reis et al., 2022)		
Seed and oil traits	High oil content – *Camelina sativa* (Kagale et al., 2016)	various genes including microRNA167A, fatty acid elongation and triacylglycerol biosynthesis genes	Enhances the nutritional and industrial value of oilseed crops
	Modified fatty acid composition - *Crambe abyssinica* (Qi et al., 2018)	*LYSOPHOSPHATIDIC ACID ACYLTRANSFERASE 2*	
	Improved seed size and pod shattering resistance - *Brassica carinata* (Raman et al., 2017, 2024)	*SHATTERPROOF 1/2 (SHP1/SHP2), FRUITFUL (Ful), MANNANASE 7(Man7)*	

Many Brassicaceae species within this continuum are cultivated or utilized for their nutritional and industrial value. For example, *C. sativa* is widely recognized as an emerging oilseed crop for food, feed, and biofuel production, with seeds containing 38–43% oil and 27–32% protein, comparable to major oilseed Brassicas (Sainger et al., 2017). Similarly, *B. carinata*, although more widely cultivated than many orphan crops, has gained attention due to its high-value oil composition suitable for biofuel production, with the potential to significantly increase hydrocarbon yield (Galloway et al., 1998; Brassica carinata – African Orphan Crops Consortium, 2013; Kumar et al., 2022; Amas et al., 2025). *Crambe hispanica* contains high erucic acid making it suitable for industrial purposes (Jeppson et al., 2019). In addition, species within the genus *Diplotaxis* (*D. muralis*, *D. tenuifolia*, *D. harra*, and *D. erucoides*) are consumed as leafy greens and are characterized by high levels of phytonutrients, fiber, minerals, amino acids, and bioactive compounds such as phenolics and glucosinolates (Vasupalli et al., 2017). Moreover, *Isatis indigotica* is known for its medicinal use due its rich bioactive compounds that have been tested for their antiviral, antibacterial and anti-inflammatory properties (Kang et al., 2020). Collectively, these crops represent a spectrum of utilization from locally important orphan crops to increasingly recognized underutilized species.

Beyond cultivated species, several Brassicaceae taxa, including wild relatives and semi-domesticated species, exhibit exceptional adaptation to extreme environmental conditions. For instance, *T. arvense*, a winter annual crucifer capable of surviving temperatures as low as −40 °C, represents a valuable model for understanding cold tolerance (Geng et al., 2021). Similarly, *L. meyenii* (maca), cultivated in high-altitude Andean environments, demonstrates tolerance to cold and high UV radiation (Quiros et al., 1996; Huarancca Reyes et al., 2019). Species such as *P. cornutum* and *S. parvula* are adapted to arid, saline, and nutrient-poor environments, providing important genetic resources for drought and salt tolerance (Dassanayake et al., 2011). Likewise, the wild species *H. incana* exhibits exceptionally high photosynthetic efficiency under intense light, making it a promising candidate for improving yield-related traits in crops (Hoang et al., 2024).

In addition to abiotic stress tolerance, Brassicaceae species also represent important sources of resistance to pests and diseases. For example, *R. indica* has demonstrated tolerance to the destructive aphid *Lipaphis erysimi* (Bandopadhyay et al., 2013), while *B. vulgaris* produces specialized metabolites such as triterpenoid saponins and glucosinolates that confer resistance to herbivorous insects (Agerbirk et al., 2003; Kuzina et al., 2009; Tlili et al., 2022). *E. sativa*, an underutilized leafy vegetable, has been reported to possess resistance to several pests and pathogens, attributed to its diverse glucosinolate profile and associated defense mechanisms (Eltalawy et al., 2025). Furthermore, *Sinapis alba* has been used as a donor for improving disease resistance in cultivated species including *B. juncea* (Singh et al., 2021). These traits highlight the importance of both cultivated and wild Brassicaceae species as reservoirs of genetic variation for improving pest and disease resistance.

## 3 Genomic resources and advances

Advancements in sequencing technologies and assembly strategies have led to significant improvements in the development of genomic resources for orphan and underutilized crops within the Brassicaceae family and their wild relatives. Currently available genomic resources range from high-resolution chromosome-level assemblies to draft genomes and plastid-only genomes (**Table 2**).

**Table 2:** Genomic resources of orphan, underutilized Brassicaceae species and their wild relatives.

Species	Genome status	Ploidy	Assembly size	No. of. genes	Sequencing tech	Source
*Armoracia rusticana*	Chromosome -level	allotetraploid (2n=4x=32)	610 mb	42,025	Multiple (Oxford, PacBio, Hi-C)	(Shen et al., 2023)
*Barbarea vulgaris*	Chromosome -level	Diploid (2n=2x=16)	246.25 mb	24,516	Multiple (PacBio, Hi-C)	(Christenhusz et al., 2025)
*Brassica carinata*	Chromosome -level, pan genome	Allotetraploid (2n=4x=34)	2.52 gb	127,427	Multiple (Oxford nanopore, Hi-C)	(Song et al., 2021; Paritosh et al., 2024)
*Camelina microcarpa*	Draft assembly	1 tetraploid (2n=4x=26) and 2 hexaploid accessions (2n=38, 2n=40)	N/A	N/A	Multiple (Oxford nanopore, PacBio)	(Mandáková et al., 2019) crusiferseq.ca
*Camelina neglecta*	Chromosome -level	Diploid (2n=12)	255.04 mb	26,595	Multiple (PacBio, Illumina NovaSeq)	(Wang et al., 2024)
*Camelina sativa*	Chromosome -level pan genome	Hexaploid (2n=6x=40)	660 mb	94,744	Multiple (PacBio, Oxford Nanopore)	(Brock et al., 2024) crusiferseq.ca
*Cardamine enshiensis*	Draft assembly	Tetraploid (2n=4x=32)	443.46 mb	52,725	PacBio, Hi-C	(Huang et al., 2021)
*Crambe abyssinica*	Plastid genome(153kb) Nuclear genome: N/A	Allohexaploid (2n=6x=90)	~3.5 gb	N/A	Illumina HiSeq for plastid and nuclear draft	(Qi et al., 2016)
*Crambe hispanica*	Chromosome level	Diploid (2n=30)	480 mb	20,402	Multiple (Pacbio, Illumina)	genome available through BMAP Phytozome genome ID: 488; NCBI taxonomy ID: 70124
*Diplotaxis erucoides*	Genome survey contigs	Diploid (2n=14)	N/A	N/A	Ion-Torrent	(Vasupalli et al., 2017)
*Diplotaxis harra*	N/A	Diploid (2n=26)	N/A	N/A	N/A	(Pignone and Martínez-Laborde, 2011)
*Diplotaxis muralis*	N/A	Diploid (2n=42)	N/A	N/A	N/A	(Pignone and Martínez-Laborde, 2011)
*Diplotaxis tenuifolia*	Draft assembly	Diploid (2n=22)	424 mb	N/A	Illumina HiSeq	ngdc.cncb.ac.cn (Pignone and Martínez-Laborde, 2011; Reis et al., 2022)
*Eruca sativa*	Draft assembly	Diploid (2n=22)	~851 mb	45,483	Illumina MiSeq and HiSeq2500	(Bell et al., 2020)
*Hirschfeldia incana*	Chromosome -level	Diploid (2n=14)	408.93 mb	54,457	Nanopore ONT sequencing, Hi-C	(Hoang et al., 2024)
*Isatis indigotica*	Chromosome-level	Diploid (2n=14)	293.88 mb	30,323	PacBio and Illumina	(Kang et al., 2020)
*Lepidium meyenii*	Chromosome -level	Octaploid (2n=8x=64)	743 mb	96,417	Illumina HiSeq	(Zhang et al., 2016; Chen et al., 2018)
*Lepidium sativum*	Draft assembly	Diploid (2n=2x=16)	380 mb	25,668	PacBio HiFi Sequel II	(Johnston et al., 2005; Patat et al., 2022)
*Orychophragmus violaceu*	Chromosome -level	Diploid (2n=24)	~1.25 gb	61,097	NovaSeq 6000, PacBio Sequel II	(Huang et al., 2023)
*Physaria fendleri*	Draft assembly	Diploid (2n=12)	273 mb	39,859	Hybrid assembly involving Illumina short reads, transcriptome data scaffolding, and reference-guided super-scaffolding based on the related Camelina laxa genome	(Johnston et al., 2024)
*Pugionium cornutum*	Chromosome -level	Unclear but polyploidy (2n=22)	550 mb	31,412	GridION, PacBio, MGISeq 2000 and Illumina	(Hu et al., 2021)
*Rorippa indica*	Low coverage genomic resource and *de novo* transcriptomics	Hexaploid (2n=6x=48)	~812 mb	N/A	Flow cytometry across 192 accessions, low coverage Illumina NovaSeq WGS for 64 accessions	(Han et al., 2025)
*Schrenkiella parvula*	Draft assembly	Diploid (2n=14)	137 mb	30,419	Single-end short read Illumina	(Dassanayake et al., 2011)
*Sinapis alba*	Chromosome-level	Diploid (2n=14)	518 mb	41,127	Pacbio and Illumina	(Yang et al., 2023)
*Sisymbrium irio*	Draft assembly	Diploid (2n=14) multiple ploidies observed (up to octoploid)	~245.6 mb	N/A	Short read Illumina	(Mancia et al., 2022) https://ngdc.cncb.ac.cn/gwh/ncbi_assembly/55107/show
*Subularia aquatica*	Plastome available but no nuclear	diploidized mesoctoploid genomes	N/A	N/A	N/A	(Dogan et al., 2022)
*Subularia monticola*	Plastome available but no nuclear	diploidized mesoctoploid genomes	N/A	N/A	N/A	(Dogan et al., 2022)
*Thlaspi arvense*	Chromosome -level	Diploid (2n=14)	527.3 mb	31,596	Illumina HiSeq, Oxford Nanopore and HiC	(Geng et al., 2021)

Chromosome-level genome assemblies are now available for a substantial number of Brassicaceae orphan and underutilized species, such as *A. rusticana* (Mandáková et al., 2019; Shen et al., 2023), *B. vulgaris* (Byrne et al., 2017; Liu et al., 2019), *B. carinata* (Song et al., 2021; Paritosh et al., 2024), *C. neglecta* (Wang et al., 2024), *C. sativa* (Brock et al., 2024), *Cardamine enshiensis* (Huang et al., 2021), *Crambe hispanica* (Phytozome genome ID: 488), *H. incana* (Hoang et al., 2024), *I. indigotica* (Kang et al., 2020), *L. meyenii* (Zhang et al., 2016; Chen et al., 2018), *O. violaceus* (Huang et al., 2023; Song, 2023)*, P. cornutum* (Hu et al., 2021; Xu and Wang, 2024), *S. alba* (Yang et al., 2023), and *T. arvense* (Geng et al., 2021). These assemblies provide a robust platform for gene discovery, synteny analysis and trait mapping. Notably, the *A. rusticana* genome has been assembled telomere-to-telomere, with complete chromosomal continuity and over 42,000 annotated genes (Shen et al., 2023). In contrast, several species, such as *D. tenuifolia* (Pignone and Martínez-Laborde, 2011; Reis et al., 2022)*, E. sativa* (Bell et al., 2020), *L. sativum* (Johnston et al., 2005; Patat et al., 2022), *P. fendleri* (Johnston et al., 2024), *S. parvula* (Dassanayake et al., 2011), and *S. irio* (Genome accession: GCA_000411075.1, Mancia et al., 2022), have draft genomes assembled to scaffold or contig levels, providing valuable data for preliminary analyses and marker discovery. However, they may lack the structural resolution required for comprehensive genomic comparisons, while *D. erucoides* (Vasupalli et al., 2017) and *R. indica* (Han et al., 2025) offer only genome survey sequences or fragmented assemblies that require further improvement. A noteworthy subset of crops, including *C. abyssinica* (Qi et al., 2016), *D. harra*, *D. muralis* (Pignone and Martínez-Laborde, 2011), *S. aquatica*, and *S. monticola* (Dogan et al., 2022), lack nuclear genome assemblies, restricting their incorporation into broader comparative or functional genomics pipelines. However, for these species, plastid genomes or cytogenetic data are available.

Pan-genome resources aim to capture genomic diversity across multiple accessions of a species. To effectively represent this diversity, especially in polyploid species where gene content and genome structure can vary widely, graph-based pan-genome models are employed (MacNish et al., 2025). These models use graph structures to encode multiple genome sequences and structural variants, enabling more precise and comprehensive analysis than conventional linear reference genomes (Sirén et al., 2021). The emergence of pan-genome and graph pan-genome initiatives has played a role in improving our understanding of the genomic diversity of orphan crops (Hu et al., 2025), such as *B. carinata* (Niu et al., 2023) and *C. sativa* (Bird et al., 2025). Notably, *B. carinata* has a pan-genome derived from 86 accessions, which enabled the identification of over 300 genes associated with agronomic and seed quality traits, including one hub gene for pod shatter resistance (*BcaFUL.B7*) (Raman et al., 2024). Similarly, the pan-genome of *C. sativa* incorporated 12 chromosome-scale assemblies and exposed sub-genome-specific diversity and core gene conservation (Bird et al., 2025). While these two species represent the most comprehensive efforts within the orphan Brassicaceae, other underutilized crops in the family are yet to reach this level of genomic resolution. Nevertheless, the increasing availability of high-quality genome assemblies and resequencing data in orphan species, such as *C. enshiensis*, *T. arvense*, and *L. meyenii*, lays a foundation for future pan-genomic initiatives (Petereit et al., 2022). While genome assemblies provide the structural framework for gene discovery and comparative analyses, understanding how these genes are regulated across tissues, developmental stages, and environmental conditions require complementary transcriptomic and epigenomic approaches.

## 4 Transcriptomic and epigenomic resources

There is an increasing availability of transcriptomic and epigenomic resources for orphan and underutilized Brassicaceae species and their wild relatives. Some species have benefited from comprehensive transcriptomic profiling, while others remain largely uncharacterized beyond genome assembly. *C. sativa* has a robust developmental transcriptome atlas spanning 12 tissues (Kagale et al., 2016), enabling high-resolution analysis of gene expression across vegetative and reproductive development. More targeted transcriptomic studies have explored cold stress responses in the ‘Suneson’ cultivar, revealing genome-wide differential expression of homeologs and a bias in sub-genome expression dynamics (Fang et al., 2023). A broader pan-transcriptomic analysis involving 48 accessions identified co-expression modules for flowering-time regulators, especially *SOC1-*like genes, and highlighted sub-genome dominance patterns (Zhang et al., 2024). These datasets are now integrated into CamRegBase, a dedicated Camelina expression database that supports network and functional analysis of regulatory pathways, particularly those involved in seed oil metabolism (Gomez-Cano et al., 2020). Similarly, *B. carinata* has seen an increase in transcriptomic resources related to abiotic stress responses, including transcriptomes under cadmium exposure that revealed 631 DEGs in shoots and 271 in roots with enrichment in ion transport and hormone biosynthesis pathways (Yang et al., 2024). Gene family studies, such as the calcium-dependent protein kinase (CPK) gene family, further indicated their roles in salt and cadmium stress signaling (Zuo et al., 2024), while fatty acid desaturases (FADs) showed transcriptional stability under stress, emphasizing their potential for improving oil quality traits (Shaheen et al., 2023).

*T. arvense* is another species with well-developed transcriptomic and epigenomic resources. Flood-tolerance transcriptomic profiling revealed thousands of DEGs associated with hypoxia response, glycolysis, and suberin biosynthesis across diverse genotypes (Combs-Giroir et al., 2024). Multi-tissue transcriptome datasets (Nunn et al., 2022) and embryo RNA-Seq data across 22 accessions (García Navarrete et al., 2022) enriched the understanding of tissue-specific gene expression and metabolic processes relevant to oil content and seed development. In a major epigenomic advance, whole-genome bisulphite sequencing (WGBS) of 207 European accessions revealed extensive natural variation in DNA methylation, with climate-associated differentially methylated regions (Galanti et al., 2022). Mobile transposable element profiling also revealed that the mobilome, the complete set of mobile genetic elements, particularly transposable elements, within a genome, contributes to both genetic and epigenetic diversity (Contreras-Garrido et al., 2024).

*S. parvula* was one of the first wild Brassicaceae species to be analyzed using comparative transcriptomics alongside *A. thaliana*, highlighting conserved and novel stress regulatory networks (Dassanayake et al., 2011; Oh et al., 2014). A recent spatiotemporal transcriptome atlas spanning 35 developmental stages and 27 salt treatments demonstrated that seedling tissues exhibit the greatest transcriptomic plasticity under salt stress, while other tissues exhibit isoform-level shifts distinct from gene-level expression changes (Wijesinghege et al., 2022). In underutilized crops such as *E. sativa*, the development of a *de novo* reference genome assembly coupled with ontogenic and postharvest transcriptomic datasets has enabled detailed characterization of sulphur metabolism and glucosinolate biosynthesis pathways (Bell et al., 2020). These studies identified key regulatory genes and expression patterns underlying metabolite accumulation and defense-related traits, providing valuable targets for crop improvement. *I. indigotica* has relatively well-developed transcriptomic resources, including *de novo* transcriptome datasets that have supported the identification of genes involved in indole, terpenoid, and phenylpropanoid biosynthesis (Chen et al., 2013). Transcriptomic analyses have been used to investigate reproductive development and to identify candidate genes associated with the regulation of flowering in *I. indigotica* (Bai et al., 2018). Emerging transcriptomic resources are also available for *S. alba*, with comparative gene expression analyses providing insights into defence-related pathways associated with resistance to fungal pathogens such as *Alternaria brassicicola* (Ahmed et al., 2024). In *B. vulgaris*, transcriptomic profiling under diamondback moth (*Plutella xylostella*) herbivory identified detoxification and secondary metabolism genes (Wei et al., 2013), while subsequent work discovered SSR markers and DEGs linked to resistance in insect-resistant genotypes (Zhang et al., 2015). In wild relatives such as *R. indica*, *de novo* transcriptome assembly and differential gene expression analyses under aphid infestation have identified candidate defense-related genes and pathways associated with insect tolerance (Bandopadhyay et al., 2024). However, compared to other Brassicaceae species, transcriptomic resources remain limited and largely restricted to targeted stress-response studies. Cross-species transcriptomic comparisons under high-light conditions have revealed *H. incana*’s regulatory dynamics in photosynthesis-associated genes, including divergence in expression of light-harvesting complexes and photosystem II-related genes, distinguishing it from relatives such as *A. thaliana*, *B. rapa*, and *B. nigra* (Garassino et al., 2022). Further, comparative transcriptomics across multiple Brassicaceae species highlighted enriched expression of genes involved in energy capture and carbon assimilation, aligning with *H. incana*’s high photosynthetic rates (Hoang et al., 2024). Earlier studies leveraging microarray and RNA-Seq under heavy metal exposure (e.g., Pb stress) identified hundreds of differentially expressed genes related to metal transport, detoxification, and antioxidant activity in both roots and shoots, offering insights into *H. incana*’s tolerance mechanisms (Auguy et al., 2016). Although direct epigenomic data, such as methylome or chromatin accessibility maps, remain unavailable, the recent chromosome-level genome assembly and sub-genome-specific gene expression analyses provide a foundation for future regulatory studies (Hoang et al., 2024). Additionally, genome-wide identification of nearly 914 resistance gene analogs (RGAs) in *H. incana* hints at lineage-specific expansions of regulatory gene families (Wu et al., 2024). As resources expand, *H. incana* holds promise for dissecting the transcriptional and epigenetic basis of stress resilience and photosynthetic efficiency in non-model Brassicaceae. Similarly, *L. meyenii* has been studied extensively using transcriptomic and proteomic approaches to explore UV-B responses (Yu et al., 2025), root development (Shang et al., 2018), and ecotype-specific secondary metabolism traits regulated by MYB and WRKY transcription factors (Chen et al., 2018). The transcriptome analysis in *P. cornutum*, uncovered thousands of DEGs linked to water transport, glutathione metabolism, and membrane transport (Wang et al., 2023; Zhao et al., 2023). Functional studies of heat shock proteins such as *Pc*Hsp70–5 further support its role in drought tolerance (Xu and Wang, 2024), while carbon metabolism and L-ascorbic acid biosynthesis under drought were also transcriptionally regulated (Yang et al., 2022).

Despite these advances, most orphan, underutilized and wild Brassicaceae species remain under-characterized in terms of transcriptomic and epigenomic data. Species such as *L. sativum, O. violaceus*, and *P. fendleri* have limited transcriptomic datasets, primarily from gene annotation or tissue-specific expression efforts (Morris et al., 2011; Johnston et al., 2024; Shi et al., 2025). In contrast, species such as *A. rusticana, C. microcarpa, C. neglecta, C. abyssinica, C. hispanica, Diplotaxis* spp.*, S. irio*, and the *Subularia* lineages have yet to benefit from high-throughput transcriptomic or methylome studies. This gap represents a significant opportunity as genome assemblies and resequencing projects grow; these species are poised for advanced regulatory studies. The identification of regulatory networks, candidate genes, and stress-responsive pathways provides a foundation for their direct domestication, as well as for trait introgression and targeted breeding in established crops.

## 5 Applications of genome resources

### 5.1 Molecular marker development

Genomic resources for orphan and underutilized Brassicaceae crops allow the development of molecular markers for various applications, including germplasm characterization, trait mapping and molecular MAS. For example, molecular markers for *C. abyssinica* were developed from *de novo*-assembled cDNA and genomic DNA, facilitating the evaluation of genetic diversity across accessions consistent with their breeding histories (Qi et al., 2016). In *D. tenuifolia*, SNP markers were developed from the genome assembly, effectively delineating it from other closely related species such as *E. sativa* (Reis et al., 2022). By providing detailed genetic information on germplasm, these markers serve as important tools for effective gene bank management and conservation.

Molecular markers derived from high-quality genomic resources have significantly accelerated trait mapping via quantitative trait locus (QTL) analysis and genome-wide association studies (GWAS). This is particularly evident in *C. sativa* and *B. carinata* where abundant genomic resources are now accessible. For instance, Chaudhary et al., 2023 identified three QTLs controlling vernalization requirements using genome-derived SNP markers, providing a target for improving flowering traits in *C. sativa*. Similarly, genomic regions linked to freezing tolerance were identified using a homozygosity mapping approach that utilized high-density SNP markers derived from long-read sequencing data (Shaikh et al., 2023). In other investigations, high-density SNP markers were used to pinpoint genomic regions controlling seed size, oil content, fatty acid composition and drought tolerance (Luo et al., 2019; Li et al., 2021). In *B. carinata*, 10K SNPs derived from genotyping-by-sequencing (GBS) were used to identify two distinct subpopulations within a germplasm collection comprising 620 lines. This revealed selective sweeps on chromosomes B03 and B08, affecting genes involved in fatty acid and secondary metabolite production (Khedikar et al., 2020). Furthermore, linkage mapping using SNP markers identified five genomic regions controlling pod shatter resistance in a biparental population (Raman et al., 2017). These studies demonstrate the utility of high-quality molecular markers in the identification of genomic regions for key agronomic traits, providing targets for molecular breeding in these crops.

Introgression breeding is considered an important strategy for introducing new alleles into cultivated *Brassica* crops. The availability of molecular markers has greatly assisted these efforts, expediting the development of improved cultivars. For instance, sequenced tag site (STS) markers were developed to track introgression of *D. erucoides* in *B. juncea* using *de novo* assembly and mapping to *Brassica* genomes (Vasupalli et al., 2017). These markers effectively classified individuals carrying the introgression, which are phenotypically indistinguishable from the recurrent parents, demonstrating their utility for alien introgression breeding. Genomic regions of *O. violaceus* were successfully transferred to *B. napus* using *B. rapa* as a bridge species (Yang et al., 2017). This resulted in the creation of introgression lines with superior silique traits and enhanced yield with further genetic mapping using high density SNP markers revealing several novel QTLs linked to these traits. In another study, blackrot resistance from *B. carinata* was successfully transferred to cauliflower (*B. oleracea*) through the use of SSR markers specific to *B. carinata* (Sharma et al., 2017). These examples illustrate the potential of molecular markers for introgression breeding, enhancing the diversity for cultivar development.

### 5.2 Comparative genomics

Conserved synteny has been retained in many orphan and underutilized Brassicaceae crops as well as their wild relatives due to their shared ancestry, enabling comparative genomics to reconstruct evolutionary trajectories and identify genes controlling adaptive traits in these species (Walden and Schranz, 2023). For example, species such as *S. parvula* and *T. arvense* have benefited from comparative genomics studies to dissect their stress tolerance and environmental adaptation (Dassanayake et al., 2011; Geng et al., 2021). Other species, such as *C. sativa* and *C. enshiensis* have been studied through comparative genomics to uncover polyploidy-derived gene duplications and sub-genome dominance (Kagale et al., 2014; Huang et al., 2021; Brock et al., 2024). By aligning the assemblies of *L. meyenii* and *O. violaceus* against the reference genomes of *A. thaliana, B. rapa* and *B. oleracea*, candidate genes involved in flowering time, secondary metabolism and oil biosynthesis were identified (Zhang et al., 2023). Comparative analyses in *B. carinata* (Amas et al., 2025), *C. neglecta* (Martin et al., 2022; Wang et al., 2024), *P. cornutum* (Hu et al., 2021; Xu and Wang, 2024), and *S. irio* (Mancia et al., 2022) have facilitated the identification and characterization of detoxification pathways, sub-genome evolution, stress adaptation, and lineage-specific expansions. Several other species, including *C. abyssinica*, *H. cardiocarpa*, and the *Diplotaxis* and *Subularia* lineages, remain less explored due to limited genomic resources. Future comparative analysis integrating high quality genome assemblies are expected to enhance our understanding of genome evolution and the functional diversity of Brassicaceae. Such cross-species comparisons not only enrich genome annotations but also reveal lineage-specific features that are valuable for trait improvement.

Comparative analyses are also defining the genomic landscape of biotic stress resistance in Brassicaceae species. Several of these crops such as *B. vulgaris*, *C. sativa*, *L. meyenii*, *S. alba, S. irio*, *S. parvula*, and *T. arvense* contain a significant number of resistance (*R*) genes including homologs of cloned disease *R* genes, which were originally identified from model and cultivated crops (Cantila et al., 2022; Dolatabadian et al., 2025). The genome-wide analysis in *B. carinata* found this species to contain the greatest number of *R* genes among the species in the triangle of U, likely reflecting its larger allotetraploid genome size as the only species combining both the B and C sub-genomes (Fredua-Agyeman et al., 2014). Notably, the B genome itself is known to harbor functionally relevant disease resistance loci; for example, B genome chromosomes B3 and B8 of *B. carinata* have been linked to blackleg resistance (Fredua-Agyeman et al., 2014). These investigations highlight the abundance of RGAs across underutilized Brassicaceae species, providing candidate loci for functional characterization and potential exploitation in breeding (Amas et al., 2025). Beyond their immediate applied value, comparative analysis of RGA repertoires across orphan and underutilized species offers insights into the evolution of disease resistance genes within the family, revealing how R gene diversity has been shaped by shared ancestry, polyploidy, and divergent selection pressures. Besides disease resistance, the genomic underpinnings of insect resistance are now being untangled using genomic data. Sequencing of the horseradish (*A. rusticana*) genome identified 279 genes involved in the biosynthesis and breakdown of glucosinolates (GSLs), a major class of secondary metabolites produced by most members of the Brassicaceae family, which have been shown to provide protection against insect pests (Shen et al., 2023). Similarly, a candidate gene (*BvUGT1*) encoding a class of saponin that deters key insect pests, including the yellow-striped flea beetle and diamondback moth, was identified in *B. vulgaris* (Augustin et al., 2012). A comprehensive analysis of these genes through comparative genomics will allow a better understanding of their evolution, which can be harnessed to improve insect resistance in key Brassicaceae crops.

### 5.3 Multi-omics and functional analysis

Linking evolutionary and comparative insights to functional trait variation increasingly relies on integrating multiple layers of biological data, highlighting the importance of multi-omics approaches. This is especially important for underutilized and orphan Brassicaceae crops if the aim is for these crops to be integrated into mainstream production systems. In *C. neglecta*, the development of chromosome-level assembly, along with transcriptomic analysis, revealed key genes and transcription factors involved in seed oil biosynthesis (Wang et al., 2024). Similarly, integrating genomic and transcriptomic data from different stages of seed development in *O. violaceus* identified *OvDGAT1–1* and *OvDGAT1–2* as candidates for regulating dihydroxy fatty acids (diOH-FAs), a type of lipid able to withstand high-temperature conditions, making it suitable for industrial use (Huang et al., 2023). Metabolomic profiling along with transcriptomic analysis revealed insights into the regulation of triacylglycerol biosynthesis in *C. sativa*, providing a basis for improving seed oil quality in this species. Moreover, proteomic analysis in maca (*L. meyenii*) identified at least 19 highly expressed proteins following high temperature stress (HTS) treatment, shedding light on the molecular responses underlying HTS tolerance in this underutilized crop (Wang et al., 2020). These multi-omics studies emphasize the power of integrative approaches for uncovering the genetic control of agronomic and stress-adaptive traits, providing valuable targets for the genetic improvement of underutilized and orphan Brassicaceae species, with potential application in major *Brassica* crops.

The availability of high-quality genome information also enables functional validation studies to further dissect the molecular mechanisms underlying trait expression. For example, the genome-guided identification of genes *PfeDGAT1* and *PfeDGAT* in *P. fendleri* enabled functional studies to understand the mechanisms underlying the production of hydroxylated fatty acids (HFA), a highly valued class of fatty acids used in the chemical industry (Parchuri et al., 2024). This work has the potential to inform the engineering of biochemical pathways for HFA production in related Brassicaceae crops, enhancing their oil quality and market value. In *C. sativa*, genome mining assisted the identification of microRNA167A, which contributes to increased seed size and improved oil quality. Genome and transcriptome analyses have assisted the functional characterization of the novel *Arabidopsis G-protein γ subunit 3 (AGG3)* revealing its involvement in seed and oil production in *C. sativa* emphasizing the role of poorly characterized genetic factors, providing novel targets for improving seed characteristics (Roy Choudhury et al., 2014). Homologs of these genes can also be mined in other related cultivated species and can be potentially harnessed to enhance seed components in these crops.

Advancements in genome sequencing have also significantly driven the deployment of sophisticated genome editing tools, such as CRISPR-Cas systems and RNA interference (RNAi), for rapid characterization of genes. For instance, modifying the *transparent testa 8* gene using CRISPR-Cas9 resulted in enhanced oil accumulation properties of yellow-seeded *C. sativa* (Cai et al., 2024). In another study, CRISPR-Cas9 was used to knockout *fatty acid desaturase2* (*FAD2*) and *reduced oleate desaturation1* (*ROD1*) genes which resulted in mutants expressing elevated oleic acid levels in *T. arvense* (Jarvis et al., 2021). Genome editing using CRISPR was also explored to modify the *fasciclin-like arabinogalactan1* (*BcFLA1*) gene controlling root hair formation in *B. carinata* (Kirchner et al., 2017). As genome editing remains to be challenging in polyploid *Brassica* species, this work may provide a valuable basis for applying similar methods to other cultivated polyploid species including *C. sativa*, *B. napus* and *B. juncea*. Moreover, engineering *C. abyssinica* to produce high erucic acid and low polyunsaturated fatty acid was successfully achieved by silencing the production of lysophosphatidic acid acyltransferase through RNAi (Qi et al., 2018). A similar approach was performed in *P. fendleri* to increase the accumulation of non-toxic lesquerolic acid by silencing several genes, including *KCS18, FAD2* and *FAD3*, using RNAi (Chen et al., 2021). These advances have greatly benefited from the availability of genome information enabling a comprehensive understanding of complex traits.

**Figure 2:**
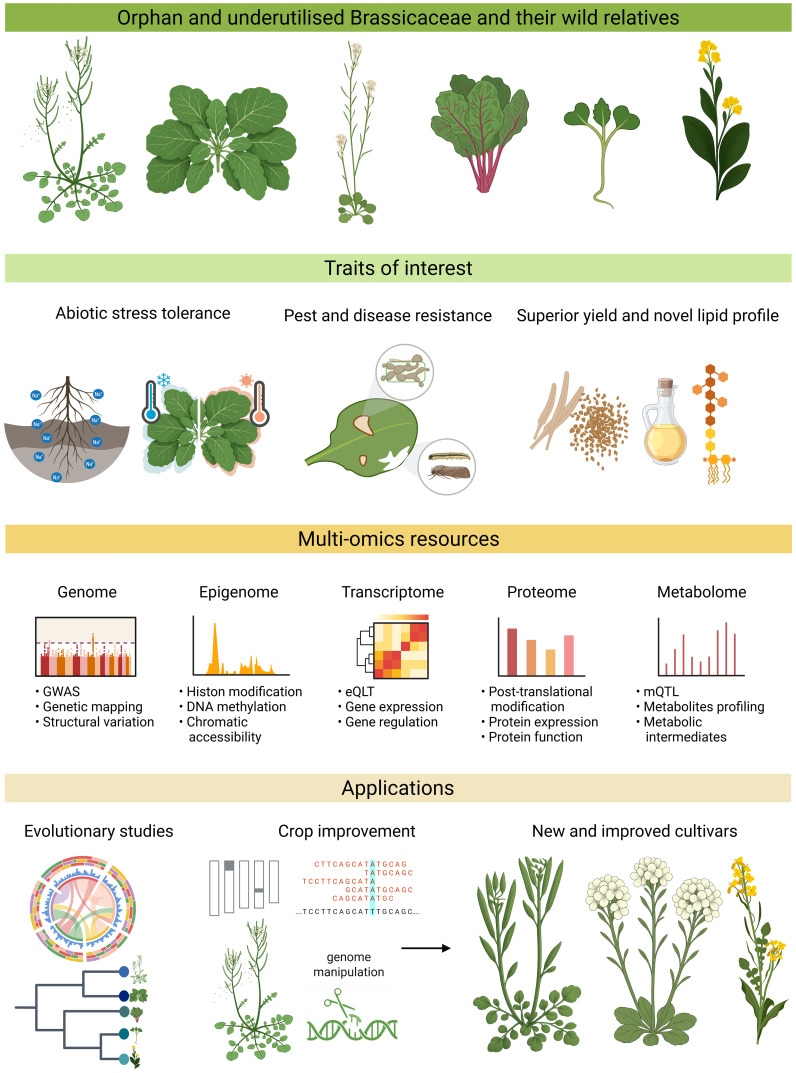
Orphan Brassicaceae crops as genetic reservoirs of key traits for crop improvement. Recent advances in genomics and multi-omics technologies have enabled the interrogation of untapped genetic diversity in these species, with immense potential to develop novel and improved cultivars. Created with BioRender.com.

However, while gene editing has been successfully demonstrated in several orphan and underutilized Brassicaceae crops, the efficiency remains lower compared to major crops due to several technical and biological bottlenecks including the inefficient transformation protocols and the inherent complexity of the genomic architecture of some species. The development of genotype-independent gene-editing approaches, advanced multi-gene editing systems and highly adaptable transformation methods will help overcome these challenges (Yuan et al., 2024). In parallel, the integration of genomic, transcriptomic, metabolomic, and proteomic datasets across these crops provides unique opportunities to connect genotype to phenotype and accelerate trait discovery. This will highlight the importance of orphan and underutilized Brassicaceae species and their wild relatives as reservoirs of novel alleles and functional gene variants that can enhance yield stability, nutritional quality, and environmental adaptability (**Figure 2**).

## 6 Challenges, opportunities, and future directions

Many orphan, underutilized, and wild Brassicaceae species still lack high-quality nuclear genome assemblies, limiting their utility for downstream analyses. For example, species such as *D. harra*, *S. aquatica*, and *S. monticola* are currently represented only by partial plastid genomes or low-coverage draft assemblies (Pignone and Martínez-Laborde, 2011; Dogan et al., 2022; Liu et al., 2022). This lack of reference genomes impedes the contextualization of transcriptomic and methylomic datasets. In species such as *O. violaceus* (Huang et al., 2023; Zhang et al., 2023) and *P. fendleri* (Johnston et al., 2024), where draft genomes are available, complex polyploid ancestry and extensive repetitive content continue to limit annotation quality and the development of pan-genome frameworks. Even when high-quality reference genomes exist, as in the polyploid *C. sativa* (2n=6x=40), the hexaploid genome comprising three closely related subgenomes presents ongoing analytical challenges due to high sequence similarity among homeologs (Brock et al., 2024). This leads to difficulties in assembly phasing, accurate gene model prediction, and distinguishing between homologous and paralogous sequences (Kagale et al., 2016; Brock et al., 2024). Moreover, transcriptomic analyses under stress conditions have revealed sub-genome-specific misregulation and allelic variation, further complicating gene expression analysis and genome annotation (Fang et al., 2023). *B. carinata* (2n=4x=34), an allotetraploid species, demonstrates sub-genome dominance, extensive gene fractionation, and significant expansion of stress-responsive and oil metabolism-related gene families, all of which contribute to highly redundant genomic regions. Such complexity can lead to fragmented assemblies or collapsed paralogs unless supported by long-read sequencing technologies with sufficient depth (Niu et al., 2023; Paritosh et al., 2024; Amas et al., 2025). Similarly, although *C. enshiensis* (2n=4x=32) has a chromosome-scale assembly (Huang et al., 2021), its genome harbors widespread segmental duplications and lineage-specific expansions, complicating automated gene-prediction pipelines. In species with large genome sizes, such as *C. abyssinica* (~3.5 Gb, allohexaploid), the prevalence of repetitive DNA and transposable elements poses substantial challenges to contiguity and scaffolding (Qi et al., 2016).

Heterozygosity poses a formidable challenge in outcrossing species such as *D. tenuifolia*, *L. sativum*, and *S. irio*, which are either facultatively or obligately outcrossing and often exhibit substantial allelic diversity (Pignone and Martínez-Laborde, 2011; Mancia et al., 2022; Patat et al., 2022). This heterozygosity can lead to mis-assembly artifacts, such as duplicated alleles, mis-collapsed haplotypes, inflated assembly size, or chimeric scaffolds, particularly when short-read data are used. Further complicating genome assembly and annotation are lineage-specific expansions of gene families involved in stress responses. In *B. carinata*, for example, the expansion of *R* genes and transcription factors requires high-resolution annotation pipelines to accurately distinguish functional genes from pseudogenes (Amas et al., 2025). Similarly, in the drought-tolerant *P. cornutum*, large-scale transcriptomic and gene family variation have been associated with its adaptation to arid and saline environments (Hu et al., 2021). In *S. parvula*, despite a compact genome (~138 Mb), adaptations to abiotic stress have led to extensive copy number variation, isoform diversity, and alternative splicing events in key gene families (Oh et al., 2014; Wijesinghege et al., 2022), necessitating precise genome annotation strategies.

Despite these technical hurdles, the genomic characterization of orphan and underutilized Brassicaceae crops and their wild relatives has substantial potential for crop diversification, nutritional enhancement, and climate resilience, yet their development faces several challenges. Limited funding and the lack of crop-specific research networks have slowed systematic breeding and functional genomics efforts. Many species still lack high-quality genome assemblies, multi-omics resources, or trait-mapping tools, which further limits the applications of emerging technologies including single cell sequencing, spatial transcriptomics, pan-epigenomics and third generation full-length transcriptomics. All these factors hinder the comprehensive exploration of the genetic potential of most orphan and underutilized species. Community-driven databases and bioinformatics platforms are important for consolidating existing data and ensuring broad accessibility. Translating orphan and underutilized species into viable crops requires targeted domestication strategies. This includes selection for desirable agronomic traits such as seed yield, oil content, stress tolerance, and reduced anti-nutritional factors. Modern approaches such as genome editing, MAS, and speed breeding can accelerate this process, enabling the rapid development of improved cultivars. Moreover, valuable alleles from orphan and underutilized species can be introgressed into existing crops to enhance resilience and nutritional quality. Hybridization and backcrossing, guided by high-resolution genetic markers or genomic selection, allow the precise transfer of stress-resilient or nutrient-rich traits from wild relatives into cultivated backgrounds.

Despite these gaps, significant opportunities exist. Cross-disciplinary research integrating agronomy, genomics, and climate modelling can accelerate the identification of stress-resilient and high-nutrient traits. Genomic and transcriptomic resources developed for species such as *C. sativa*, *B. carinata*, and *T. arvense* illustrate the transformative potential of genomics-enabled breeding, including MAS, candidate gene identification, and genome-editing approaches. These tools not only facilitate rapid domestication and trait improvement but also allow the transfer of valuable alleles from wild relatives into cultivated crops, enhancing resilience against biotic and abiotic stresses. Furthermore, the identification of key genes in several orphan and underutilized Brassicaceae crops, such as those mentioned above (i.e. *C. sativa*, *T. arvense* and *P. fendleri*), presents exciting opportunities to rapidly domesticate these crops through *de novo* domestication using genome editing techniques such as CRISPR-Cas systems. This is further supported by the development of efficient genetic transformation systems in these crops, enabling rapid generation of transgenic lines (Wang et al., 2024; Rezaeva et al., 2025). *De novo* domestication can lead to the creation of new *Brassica* cultivars with superior agronomic characteristics comparable to major crops. Recent successes illustrate the power of these approaches. Field pennycress (*T. arvense*) has been effectively *de novo* domesticated through CRISPR-Cas9 multiplexing, with stacked mutations in *FAE1*, *MYB28*, *MYC3*, *FAD2*, *ROD1*, and *TT8* collectively producing ‘double-low’ canola-like varieties with reduced weediness and improved fatty acid profiles (Jarvis et al., 2021; Gautam et al., 2026). *Lepidium campestre* (field cress) represents another active domestication program, where QTL mapping for pod shattering, combined with established transformation protocols, is accelerating its development as a perennial oilseed catch crop (Desta et al., 2020). Beyond Brassicaceae, *Gynandropsis gynandra* (Cleomaceae), a nutritious African leafy vegetable in the sister family to Brassicaceae, is emerging as a domestication target, with genomic resources and agronomic improvement strategies now underway (Zenchyzen and Hall, 2026).

Future research should focus on expanding genome assemblies, developing pan-genomes, and generating multi-omics datasets across under-characterized species. This can be assisted by artificial intelligence (AI), which can handle massive and complicated datasets (Yetgin, 2025). For example, AI tools such as AlphaGenome (Avsec et al., 2026) can process thousands of genomic features, including gene models, transcription initiation sites, chromatin contact maps, and transcription-binding sites, among others, facilitating comprehensive genome analysis in a streamlined process. Furthermore, AI tools can derive genotype-phenotype relationships from limited datasets and leverage information from well-studied crops to predict useful traits in underutilized species (MacNish et al., 2025). This is further supported by the development of high-throughput phenotyping (HTPs) methods that provide scalable phenotypic datasets across environments that are crucial for robust selection (Danilevicz et al., 2025). These advancements are complemented by speed breeding technologies that dramatically decrease generation time, resulting in accelerated fixation of desirable alleles (Watson et al., 2018). These improvements have the potential to efficiently integrate orphan and underutilized Brassicaceae crops and their wild relatives in crop breeding, allowing the development of climate-resilient, nutrient-dense alternatives, reducing reliance on a narrow set of staple crops while enhancing global food security. These technologies are particularly relevant for developing countries and low-resource breeding programmes, where orphan and underutilized crops are often important for smallholder livelihoods but receive limited research investment. Recent orphan-crop genomics initiatives have shown that open-access genomic resources, capacity building, and shared breeding platforms can support crop improvement in regions where conventional breeding infrastructure is limited (Jamnadass et al., 2020; Gelaye et al., 2025). Similarly, low-cost or portable high-throughput phenotyping, UAV-based imaging, AI-assisted trait prediction, and speed breeding can reduce reliance on expensive field trials and shorten breeding cycles, making selection for drought tolerance, pest resistance, nutritional quality, and yield stability more feasible in resource-limited settings (Talabi et al., 2022). When combined with regional germplasm collections and farmer-participatory breeding, these approaches provide a practical route for developing locally adapted, climate-resilient cultivars in developing countries.

In conclusion, orphan and underutilized Brassicaceae crops and their wild relatives represent a largely untapped genetic resource with the potential to reshape cropping systems. However, the extent of their utilization depends on their economic value, and continuous investment will be key in realizing their potential for crop improvement and agricultural production. Despite these challenges, significant developments in the genomic characterization of these species are closing the knowledge gaps that allows their gradual integration into mainstream agricultural systems. Strategic introgression of traits through emerging technologies and methodologies enables rapid conversion of these species into productive crops and the enrichment of existing cultivars. Harnessing their genetic diversity can support crop diversification, improve nutrition, and strengthen resilience in the face of climate change, positioning these species as valuable contributors to sustainable and resilient agricultural systems worldwide.
